# *Ralstonia syzygii*, the Blood Disease Bacterium and Some Asian *R. solanacearum* Strains Form a Single Genomic Species Despite Divergent Lifestyles

**DOI:** 10.1371/journal.pone.0024356

**Published:** 2011-09-08

**Authors:** Benoît Remenant, Jean-Charles de Cambiaire, Gilles Cellier, Jonathan M. Jacobs, Sophie Mangenot, Valérie Barbe, Aurélie Lajus, David Vallenet, Claudine Medigue, Mark Fegan, Caitilyn Allen, Philippe Prior

**Affiliations:** 1 Peuplements Végétaux et Bioagresseurs en Milieu Tropical (UMR PVBMT), INRA-CIRAD, Saint Pierre, La Réunion, France; 2 Peuplements Végétaux et Bioagresseurs en Milieu Tropical (UMR PVBMT), CIRAD, Saint Pierre, La Réunion, France; 3 Unité Ravageurs et Agents Pathogènes Tropicaux, Agence Nationale de Sécurité Sanitaire, Laboratoire de la Santé des Végétaux, Saint Pierre, La Réunion, France; 4 Department of Plant Pathology, University of Wisconsin-Madison, Madison, Wisconsin, United States of America; 5 Laboratoire d'Analyse Bioinformatique en Génomique et Métabolisme, CNRS-UMR 8030, Evry, France; 6 Institut de Génomique, Genoscope, Commissariat à l'Energie Atomique (CEA) Direction des Sciences du Vivant, Evry, France; 7 Department of Primary Industries, Biosciences Research Division, Attwood, Victoria, Australia; University of Wisconsin-Milwaukee, United States of America

## Abstract

The *Ralstonia solanacearum* species complex includes *R. solanacearum*, *R. syzygii*, and the Blood Disease Bacterium (BDB). All colonize plant xylem vessels and cause wilt diseases, but with significant biological differences. *R. solanacearum* is a soilborne bacterium that infects the roots of a broad range of plants. *R. syzygii* causes Sumatra disease of clove trees and is actively transmitted by cercopoid insects. BDB is also pathogenic to a single host, banana, and is transmitted by pollinating insects. Sequencing and DNA-DNA hybridization studies indicated that despite their phenotypic differences, these three plant pathogens are actually very closely related, falling into the Phylotype IV subgroup of the *R. solanacearum* species complex. To better understand the relationships among these bacteria, we sequenced and annotated the genomes of *R. syzygii* strain R24 and BDB strain R229. These genomes were compared to strain PSI07, a closely related Phylotype IV tomato isolate of *R. solanacearum*, and to five additional *R. solanacearum* genomes. Whole-genome comparisons confirmed previous phylogenetic results: the three phylotype IV strains share more and larger syntenic regions with each other than with other *R. solanacearum* strains. Furthermore, the genetic distances between strains, assessed by an *in-silico* equivalent of DNA-DNA hybridization, unambiguously showed that phylotype IV strains of BDB, *R. syzygii* and *R. solanacearum* form one genomic species. Based on these comprehensive data we propose a revision of the taxonomy of the *R. solanacearum* species complex. The BDB and *R. syzygii* genomes encoded no obvious unique metabolic capacities and contained no evidence of horizontal gene transfer from bacteria occupying similar niches. Genes specific to *R. syzygii* and BDB were almost all of unknown function or extrachromosomal origin. Thus, the pathogenic life-styles of these organisms are more probably due to ecological adaptation and genomic convergence during vertical evolution than to the acquisition of DNA by horizontal transfer.

## Introduction

The *Ralstonia solanacearum* species complex consists of four phylogenetically distinct major lineages, named phylotypes [1]. Each phylotype contains strains primarily isolated from specific geographic areas: phylotype I strains are from Asia; phylotype II are from the Americas; phylotype III are from Africa; and phylotype IV are from Indonesia, Japan, Australia, and the Philippines. Comparative Genomic Hybridization (CGH) microarrays and whole genome sequence comparisons have confirmed the robustness of this classification scheme [2], [3]. The phylotype system synthesizes the extraordinary degree of heterogeneity found within this group of wilt-causing plant pathogens, and supports Hayward's suggestion that the evolutionary origins of *R. solanacearum* predated the geological separation of the continents [4]. Using 16S rRNA gene sequences, Taghavi *et al.* [5] showed that the banana Blood Disease Bacterium (BDB) and *Ralstonia syzygii* (formerly known as *Pseudomonas syzygii*) are very closely related to *R. solanacearum*. Within the *R. solanacearum* group, BDB and *R. syzygii* are most similar to the *R. solanacearum* strains from Indonesia, and thus belong to phylotype IV [6]. The four phylotypes encompass three different species, thereby justifying the use of the term “species complex”, defined as a cluster of closely related isolates whose individual members may represent more than one species [7].

BDB, the causative agent of blood disease, a widespread and severe wilt disease of banana and plantain in Indonesia [8], was originally named *Pseudomonas celebensis* [9]. However, the original culture deposited as the type strain of *P. celebensis* no longer exists so the name is taxonomically invalid [10]. The symptoms of blood disease are similar to those induced by *R. solanacearum* strains that cause the Moko disease of banana, which originated in Central America (phylotypes IIA-6, IIB-3, and IIB-4, historically known as Race 2). However, unlike Moko disease causing strains, BDB is not pathogenic to *Heliconia* spp. in the wild, nor to solanaceous hosts following artificial inoculation [11]. Infection of banana mats by BDB may originate from contaminated soil or water, but epidemics are usually due to nonspecific mechanical transmission by insects visiting banana flowers [12]. Blood disease symptoms include yellowing and wilting of the mature leaves, vascular discoloration, bacterial ooze, and the eponymous typical reddish-brown fruit rot characteristic of this disease [13].

*R. syzygii* causes Sumatra disease of cloves and has been responsible for the widespread death of clove trees in Sumatra and Western Java [14]. External symptoms of Sumatra disease develop after a long incubation period (>200 days), and include yellowing, followed by sudden loss of leaves from the leader tips or from a lateral branch high in the tree crown. In a few months, lower branches are affected, leading eventually to the death of the tree [15]. *R. syzygii* multiplies in the xylem vessels of clove trees and in its vector, xylem-feeding *Hindola* spittlebugs. These tube-building cercopoid insects actively and specifically transmit *R. syzygii* to healthy clove trees [16], [17].

Despite the close phylogenetic relationship among strains of *R. solanacearum,* BDB and *R. syzygii* in phylotype IV, their lifestyles are remarkably different. *R. solanacearum* is a highly heterogeneous species with a broad host range that spans monocots such as banana and ginger, and dicots including many solanaceous plants. The pathogen is typically soilborne, with the exception of a subset of phylotype II banana strains that can be nonspecifically transmitted by pollinating insects. *R. solanacearum* usually enters host roots through wounds or natural openings, and aggressively colonizes the xylem vessels, spreading through the plant and reaching population densities as high as 10^10^ CFU/ml xylem fluid [18]. Bacterial wilt disease symptoms include stunting and chlorosis; rapid, often unilateral wilting of leaves or stems; vascular browning, and often death. The previously-sequenced phylotype IV *R. solanacearum* strain, PSI07, was isolated from a wilting tomato and causes typical bacterial wilt symptoms [6].

To date, the genomes of seven *R. solanacearum* strains have been sequenced, representing considerable diversity and all four phylotypes in the species complex [3], [19], [20], [21]. However, most of these strains have similar lifestyles: they are soilborne with relatively broad host ranges, and all but two were originally isolated from tomato plants. To understand the striking biological differences among strains in phylotype IV, we sequenced and annotated the genomes of a BDB strain, R229, and a *R. syzygii* strain, R24. We used a comparative genomic analysis approach drawing on previously-published *R. solanacearum* genome sequences, with particular attention to phylotype IV tomato strain PSI07, but also considering distant bacteria sharing the same life-style. Our goal was to correlate genomic features of the three species with their distinct host ranges, symptomatology, and modes of transmission, and confirm the phylogenetic relationships among them.

Our comparative analyses offer additional evidence supporting division of the *R. solanacearum* species complex into three different species, as previously proposed [3].

## Results and Discussion

### Main features of the newly sequenced genomes

The genomes of BDB and *R. syzygii* share many characteristics with the seven previously sequenced *R. solanacearum* genomes, including a two-replicon genome structure consisting of a chromosome and a megaplasmid. Further, the guanine plus cytosine content, the single rRNA operon, and the number of tRNA genes are also quite similar in the three phylotype IV genomes (Table 1). Additional data on genome characteristics, including those of strains GMI1000, Molk2, IPO1609, CFBP2957 and CMR15, are provided in Table S1.

**Table 1:** Main genome features of strains from phylotype IV of the *R. solanacearum* species complex.

	Origin	Chr. length	Mpl. length	GC%	#CDS	rRNA op	tRNA	Ref. genome
*R.solanacearum* PSI07	Indonesia	3,508,632	2,084,845	66.3%	5247	1	49	[3]
BDB strain R229	Indonesia	3,574,388	1,584,610	66.5%	4629	1	45	This study
*R. syzygii* R24	Indonesia	3,680,625	1,743,366	65.9%	4867	2	50	This study

(Origin  =  geographical origin, Chr.  =  chromosome, Mpl.  =  Megaplamsid, GC% =  Guanine and cytosine content, #CDS  =  number of coding sequences, rRNA op  =  number of rRNA operons, tRNA  =  number of tRNA genes).

In terms of length, the chromosomes of BDB and *R. syzygii* are slightly larger than the chromosome of PSI07, but resemble the chromosome lengths reported for other *R. solanacearum* strains, which average 3.6 Mb (See [3]). On the other hand, the megaplasmids of *R. syzygii* and especially BDB are significantly smaller than those of PSI07 and other *R. solanacearum* strains. Thus, the total genome of BDB is the smallest yet sequenced within the species complex; the relatively small size of the megaplasmid in several strains of BDB and *R syzygii* strains was previously observed using pulsed field gel electrophoresis analysis (P. Prior, M. C. Lee and M. Fegan, unpublished data). Reductive evolution is common in obligate parasites and endosymbionts [22], [23], and is often due to gene deletion or decay [24], [25]. The reduced size of the megaplasmids in BDB and *R. syzygii* could be a consequence of their narrow host range and specialized life-style.

**Figure 1:**
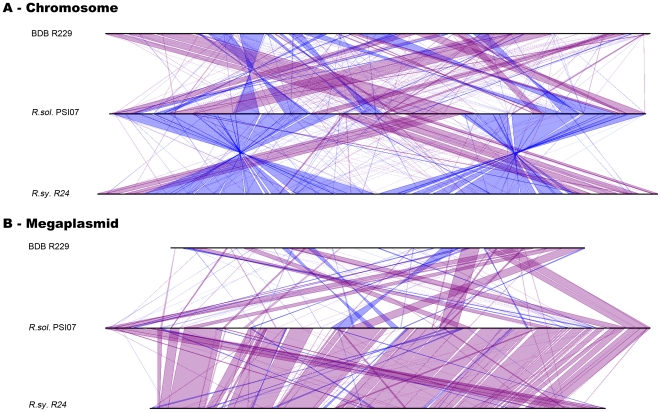
Genome alignment. Conserved synteny lineplot between *R. solanacearum* PSI07 (as reference), *R. syzygii* R24 and BDB R229, in the chromosome (1A) and the megaplasmid (1B). Strand inversions are lined in blue.

On the chromosome, which is the more conserved of the two replicons, only 70% of the CDS belonging to PSI07 were in synteny with the BDB genome, but 86% of PSI07 CDS were syntenic with the *R. syzygii* genome (see Figure 1A and Table S2A). In comparison, the degree of chromosomal synteny between PSI07 and the other sequenced *R. solanacearum* strains was between 80 and 85% of the chromosome [3]. On the megaplasmid (Figure 1B and Table S2A), this difference is amplified since only 40% of the CDS on the PSI07 megaplasmid were syntenic with the BDB megaplasmid. Synteny between megaplasmids of PSI07 and other *R. solanacearum* strains varies between 65 and 70%. This genome structure result is in conflict with the nearly identical endoglucanase (*egl*) gene sequences that placed BDB and PSI07 together in the same sequevar [6]. Large pieces of the PSI07 chromosomal sequence are found in the megaplasmid of BDB, and vice versa (Table S2B). This large-scale rearrangement was not observed in any previously sequenced strains in the species complex, and further experimental validation is required to confirm the *in-silico* assembly. When synteny is analyzed on the genome as a whole, PSI07 and BDB are the most syntenic strains in the species complex. Thus, the lack of synteny between these two strains when analyzing each replicon separately seems to be due to a recent large rearrangement between replicons in BDB R229, and not to an ancient divergence.

With respect to *R. syzygii*, the high number of CDS in synteny and the high average number of CDS per syntenic region confirmed this group's phylogenetic position in phylotype IV of the species complex. Comparative functional analysis of gene content in BDB and *R. syzygii* with that of strain PSI07 will help determine why these three closely-related organisms have such different pathogenic behaviors.

### Comparative analysis of gene content within phylotype IV strains of the species complex

**Figure 2:**
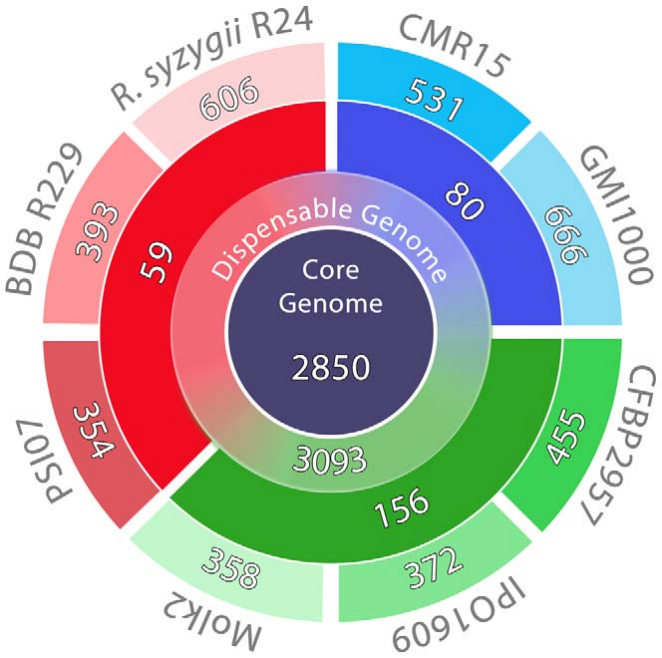
Number of genes in the species complex pan-genome. From inside to outside: Core Genome, Dispensable Genome, Specific Genome at the phylotype level (blue: phylotype I and III; green: phylotype II; red: phylotype IV), and Specific Genome at the strain level.

The pan-genome of the *R. solanacearum* species complex is defined as the set of genes present in the collective genomes of the group of organisms [3]. Sequencing BDB and *R. syzygii* adds many new genes to this known pan-genome (Figure 2). Among the 606 newly detected CDS in *R. syzygii*, 86% encoded putative proteins of unknown function, 0.7% encoded apparent phage proteins and 2.2% encoded transposases. Only 393 new CDS were detected in the genome sequence of BDB, of which 76% encoded proteins of unknown function, 18% encoded phage proteins and 3.7% encoded transposases. Previous comparative genomic analyses have detected two particular features within the genome of PSI07: a small plasmid, pRSI13, and the *rhi* operon, encoding the anti-mitotic rhizoxin toxin [3]. pRSI13 is absent from BDB and *R. syzygii,* suggesting that this plasmid was recently acquired by PSI07. However, the rhizoxin operon was detected in BDB and has also been found in the phylotype II *R. solanacearum* strain CFBP2957 [3]. The presence of the *rhi* operon in such phylogenetically distinct lineages suggests that it may have been introduced into a common ancestor of phylotype II and phylotype IV by horizontal gene transfer. Strains in phylotype IIB and *R. syzygii* may have lost the region during the course of evolution. The genomes of more strains belonging to phylotypes IV and II need to be investigated in order to understand the history of the *rhi* operon in the descent of *R. solanacearum*. Functional analysis of the fitness and relative competitiveness of a *rhi-* strain may identify the biological role of the toxin.

**Figure 3:**
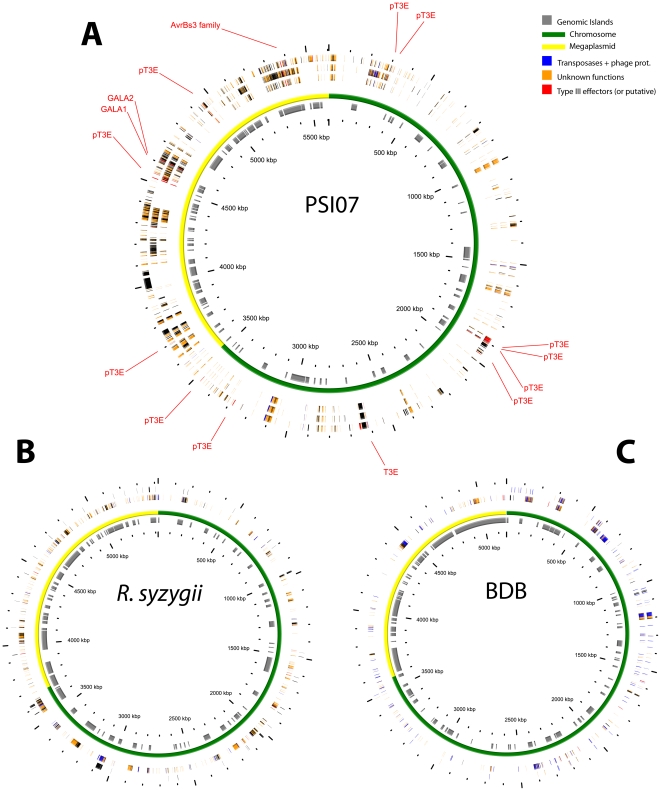
Localization of specific genes in the PSI07, *R. syzygii* and the BDB genomes. For each figure, the two inner circles represent the localization of genomic islands and a graphical representation of the genome (green = chromosome, yellow = megaplasmid) A: Genome of *R. solanacearum* strain PSI07. From inside to outside: (1) PSI07 CDS absent in both BDB and *R. syzygii*, (2) PSI07 CDS absent in *R. syzygii* only, (3) PSI07 CDS absent in the BDB only. B: Genome of *R. syzygii* strain R229. From inside to outside: (1) *R. syzygii* CDS absent in PSI07, (2) *R. syzygii* CDS absent in PSI07 but present in BDB. C: Genome of the BDB strain R24. From inside to outside: (1) BDB CDS absent in PSI07, (2) BDB CDS absent in PSI07 but present in *R. syzygii*.

Figure 3 shows the location of specific genes and genomic islands present on the chromosomes and the megaplasmids of PSI07, *R. syzygii* and BDB, with each strain compared to the other two. Genomic islands are parts of genomes that display evidence of horizontal acquisition. They have a minimal length of 5 kb and contain CDSs with no Bi-directional Best Hit, and no synteny with genomes of related organisms. Genes were assigned as being of extrachromosomal origin (phages, transposons, etc.), transcriptional regulators, type III effectors (T3E) and proteins of unknown function. In all three genomes, the density of genomic islands is two-fold greater on the megaplasmids compared with the chromosomes, which is consistent with previously studied genomes of other *R. solanacearum* strains [3]. Similarly, greater than 60% of the strain-specific genes in strain PSI07 are located on the megaplasmid (Figure 3A). However, these genes are not always members of a genomic island. Almost 800 genes present in PSI07 are not found in BDB. Similarly, 500 genes present in PSI07 are absent from both strains. In each of the phylotype IV genomes, a majority of strain-specific genes encode for proteins of unknown function (62% of strain-specific genes code for proteins of unknown function in the genomes of PSI07 and BDB). *R. syzygii* strain R24 has a higher number of strain-specific genes than BDB and PSI07 (Figure 3C), with almost 70% encoding proteins of unknown function.

Compared to PSI07, BDB (Figure 3B) has a relatively smaller number of strain-specific genes (*i.e.* only 680 BDB genes have no orthologs in the PSI07 genome). Most of them encode proteins of unknown function (65%) or proteins of phage and transposon origin (26%). In contrast, less than 8% of *R. syzygii*-specific genes are from phage or transposon origin. The BDB genome appears to have been remarkably porous, carrying much more unique genetic information from phages than *R. syzygii* or any sequenced *R. solanacearum* genome. Horizontal gene transfer mediated by prophage is known to be an important mechanism for bacterial genome evolution by mediating the acquisition and loss of ordered genes. In general, the gain of genetic material is ephemeral because such genes are easily lost by prophage genome decay or excision [26], but given the density of apparently horizontally-transferred genetic material in the BDB genome, this mechanism may have significantly shaped the pathogen, particularly with respect to adaptation to environmental constraints and host specificity [27]. Furthermore, insertion of a phage or any mobile genetic elements (*e.g.* insertion sequences) can also have a localized impact by inactivating individual genes or operons. Despite this unusually large amount of phage DNA material compared with the genomes of other strains in the species complex, BDB has a smaller genome than other sequenced representatives. Therefore it appears that BDB has lost many genes during descent from the common ancestor in comparison to other *R. solanacearum* strains. It is possible that this represents the first step of genome decay, which has been observed in many other pathogens [28] and may result in specialization on a particular host. As an example, the fastidious xylem-limited bacterium *X. fastidiosa* has experienced a genome reduction in comparison to other *Xanthomonadaceae* with which it shares a common ancestor [29]. Song *et al.* [30] have observed a similar type of evolution in the early stage of genome-evolution in *Burkholderia mallei*, where large rearrangements and deletions are associated with a high number of mobile elements.

Like many pathogenic bacteria, *R. solanacearum* uses a type III secretion system (T3SS) to deliver effector proteins, which manipulate the host physiology to increase pathogen success. *R. solanacearum* mutants lacking this secretion system are dramatically reduced in virulence [31], [32], [33]. Loss of individual effectors generally has little or no effect on virulence, but in the repertoire of T3 effectors of each strain is believed to shape host range and determine aggressiveness of the strain. The T3SS is apparently intact in BDB and *R. syzygii*, and the overall numbers of putative T3 effectors and transcription regulators are similar in PSI07 and BDB. However, the repertoire of T3 effectors is quite distinct in each strain: among the 65 known effectors or putative effectors in PSI07 [3], 13 were absent from *R. syzygii,* but only 4 are absent from BDB (Table S3). Because these T3 effectors play key biological roles in triggering host recognition and suppressing host defenses, the loss of T3 effectors may be critical evolutionary events that could explain the adaptation to insect transmission and narrow host specificity of BDB and *R. syzygii*.

*R. solanacearum* species complex strains vary in their capacity for dissimilatory nitrate metabolism (NO_3_ → NO_2_ → NO → N_2_O → N_2_). Hayward found that phylotypes I and III strains can use nitrate anaerobically as a terminal electron acceptor [34]. Consistent with this physiological result, the genome of the phylotype I strain GMI1000 encodes a full dissimilatory pathway and genes in this pathway are highly expressed when the bacterium is growing in tomato plants [19][Jacobs, Babujee, Meng and Allen, unpublished results]. Furthermore, for anaerobic growth in culture and for full virulence on tomato, *R. solanacearum* strain GMI1000 requires a functional nitrous oxide reductase (NosZ), which catalyzes the last step in this pathway by reducing nitrous oxide to nitrogen gas [Gonzalez, Dalsing, Jacobs and Allen, *unpublished results*]. In contrast, the tested phylotype II and IV strains can only perform micro-aerobic respiration by reducing nitrate to nitrous oxide [34]. This is likely because the *nosZ* gene is notably absent from the genomes of phylotype II strains UW551, IPO1609, and Molk2 and from phylotype IV strain PSI07 [3], [20]. However, neither BDB nor *R. syzygii* have the nitrate reductase genes that encode the first step in the dissimilatory pathway (*narG, narI*, and *narJ*), even though these genes are present in all other *R. solanacearum* species complex strains sequenced to date (Table S4 [35]). The apparent complete absence of dissimilatory nitrate metabolism may explain why both BDB and *R. syzygii* are fastidious and slow-growing in culture. It remains to be determined, however, how these two successful pathogens effectively colonize plant vasculature without a core metabolic pathway that is conserved in all the members of the species complex and could enable respiration in the hypoxic xylem vessels. We speculate that some conditions in their specific environment or hosts render this capacity superfluous. It should be noted that all three phylotype IV strains, along with all other species complex strains sequenced to date, produce a high-affinity *cbb3*-type cytochrome *c* oxidase encoded by the *ccoN* operon; this oxidase is required for growth under low-oxygen conditions and for full bacterial wilt virulence [36].

Both BDB and *R. syzygii* lack swimming motility [37], [38], so it is unsurprising that flagellar protein genes like *fliC* and *fliT* are absent from their genomes (Table S3). However, both strains have retained the energy taxis sensor Aer2. Possibly this protein, which senses cellular metabolic status and is required for aerotaxis and full virulence in the motile phylotype II strain K60 [39], has been modified in BDB and *R. syzygii* to transmit information about the cell's energy level to a different signaling pathway.

Toxin efflux pumps, which enable bacteria to excrete antibiotics and plant antimicrobial phytoalexins, are highly expressed by *R. solanacearum* during tomato pathogenesis and at least two are required for growth in the presence of diverse toxins and for full bacterial wilt virulence [40], [41]. However, genes for two toxin efflux pumps, AcrF and MexD, are absent from all three phylotype IV genomes (Table S3). A third, DinF, is present only in PSI07. Minimal inhibitory concentration (MIC) experiments could determine if the toxin-resistance profiles of these strains differ from those of *R. solanacearum* strains with a larger palette of toxin efflux pump genes. For examples, the DinF toxin efflux pump confers significant tolerance for the solanaceaous phytoalexins esculetin and tomatine [41]. If the absence of DinF reduces the ability of BDB and *R. syzygii* to tolerate specific plant-produced antimicrobials, this could help explain their narrow host range.

In the same way that GMI1000, the first sequenced strain of *R. solanacearum*, was not representative of the diversity in the species complex, we can anticipate that BDB R229 and *R. syzygii* R24 genomes may not be representative of these groups. In fact, very little is known about the genetic or phenotypic diversity of these bacteria at the isolate level. According to endoglucanase gene sequencing and Pulsed Field Gel electrophoresis, there appears to be little diversity among isolates of BDB [42] but there is a greater diversity among isolates of *R. syzygii* (M. Fegan, personal communication).

### Comparative analysis with bacteria sharing the same life-style

Comparative genome analysis between BDB and *R. syzygii* and their closest relatives offers no obvious explanation for their particular life-styles. However, bacteria in the *R. solanacearum* species complex can acquire large amount of exogenous DNA relatively easily [43], [44]. Thus, DNA obtained by horizontal gene transfer from more distant relatives with similar life-styles, could help explain the unique pathogenic traits of these strains. Comparative genomic analysis with these organisms may provide some answers to this question.

**Insect transmission:** ***Ralstonia syzygii*** **and** ***Xylella fastidiosa.*** Both *R. syzygii* and *Xylella fastidiosa* are fastidious xylem limited bacteria (XLB). They are both endophytic bacterial parasites that live exclusively in plant xylem cells or tracheary elements [16]. These microorganisms also both move between host plants by multiplying in sucking insects that feed on xylem fluid. *X. fastidiosa* was the first plant pathogen to be sequenced [45] and its pathogenic traits are better understood than those of *R. syzygii*. Proteins involved in bacterial adhesion, such as adhesins and hemagglutinins, play an important role in the development of *X. fastidiosa* plant pathogenicity as well as in the bacterium's ability to persist and multiply in its insect vector [46], [47], [48]. A search for genomic regions that are present in both *R. syzygii* and the seven available *X. fastidiosa* genomes, but absent from *R. solanacearum* genomes revealed 11 genes, one of which encodes an uncharacterized putative hemagglutinin-like protein. It is unlikely that this gene was horizontally transferred between *R. syzygii* and *X. fastidiosa* since it is not involved in a synteny group. However, it is noteworthy that the genome of *R. syzygii* encodes substantially more adhesins or hemagglutinins than other *R. solanacearum* (26 in *R. syzygii* versus 9 to 15 in other *R. solanacearum*) (Table S5). Experiments are needed to determine if these putative adhesins are functional, as some were annotated as gene fragments. However, adhesin fragments may retain function as the relevant protein domains can operate independently [49]. Conversely, any change in the amino-acid sequence coding domain that affects the adhesion property might drastically reduce the protein function [50].

Chitin is a major potential carbon source for bacterial growth in insects, therefore it is speculated that chitinases are important in the life-cycle of *X. fastidiosa*, particularly *chiA* [51]. No orthologs for the *chiA* gene were identified in the genome of *R. syzygii* strain R24. However, *R. syzygii* encodes two enzymes predicted to degrade chitin: a putative chitinase, and a putative chitin deacetylase. These are also present in other *R. solanacearum* genomes. Biochemical and mutational analyses could confirm these enzymatic activities and determine their biological roles in the interaction with the insect vector.

**Infecting bananas: Blood Disease Bacterium,** ***R. solanacearum*** **Molk 2 and** ***Xanthomonas campestris*** **pv.** ***musacearum.*** BDB and Moko disease Phylotype II strains of *R. solanacearum* phylotype II cause similar symptoms on banana and plantain. However, most Moko strains are also pathogenic on tomato (*Solanum lycopersicon*), unlike BDB. Moko disease-causing strains and BDB are believed to have independently evolved pathogenicity on wild *Heliconia* species in Central America and wild *Musa* species in Indonesia, respectively [5], [52]. Among the nine genes uniquely present in both phylotype II banana strain Molk2 [21] and BDB, none have been previously associated with virulence or pathogenicity. Two of them are fragments of putative type III effectors, but complete versions of these effectors are present in other *R. solanacearum* genomes. It would be useful to see if these truncated T3 effectors can alter host range of other *R. solanacearum* strains.

*Xanthomonas vasicola (formerly known as X. campestris* pv. *musacearum* [53]) recently emerged in East Africa, where it is causing a devastating epidemic of *Xanthomonas* banana wilt disease [54], [55]. Comparing the genome of *X. vasicola* to those of closely related Xanthomonads not pathogenic to banana identified major differences in lipopolysaccharide synthesis and type IV pili, but the biological consequences of these changes have yet to be determined [56]. A comparison of the *X. vasicola* and BDB genomes to those of other *R. solanacearum* strains identified nine genes that were present only in BDB and *X. vasicola* (identity ≥40%). None of these genes are involved in a synteny group and all code for proteins of unknown function. This suggests that pathogenicity to banana resulted from independent ecological convergence events in *X. vasicola* and BDB, rather than from any gene transfer conferring the ability to infect banana. A first step in testing this hypothesis would be functional analysis of the nine “banana-specific” CDS to confirm that they are not required for virulence on *Musaceae*.

### Taxonomy in the species complex and proposal of new species

**Figure 4:**
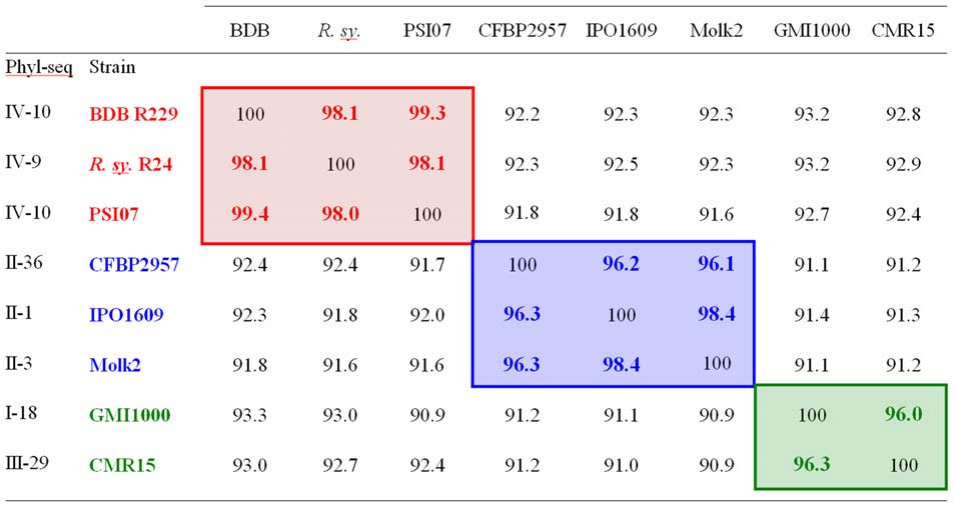
Average Nucleotide Identity (ANI) pairwise comparisons among sequenced strains in the *R. solanacearum* species complex. ANI was calculated using the method of Konstaninidis and Tiedje [57]. Strains with ANI values over 95% are considered to belong in the same species. Red text denotes strains belonging to proposed *Ralstonia haywardii* nov. species, Blue text denotes strains proposed to be retained in *Ralstonia solanacearum,* Green text denotes strains belonging to proposed *Ralstonia sequeirae* nov. species.

Based on computation of genomic distances between sequenced *R. solanacearum* strains, we previously suggested that the *R. solanacearum* species complex should be restructured into three different species: one containing phylotypes I and III, a second containing phylotype II, and a third containing *R. solanacearum* strains from phylotype IV [3]. This proposal was further supported by CGH microarray analysis of 51 *R. solanacearum* strains from all four phylotypes, but the lack of genomic data on non-*R. solanacearum* phylotype IV strains constrained this idea. Sequencing of BDB and *R. syzygii* genomes allowed us to integrate data from all described species within the species complex. We used Average Nucleotide Identity (ANI) analyses [57], to supplement previous data [3] and calculated genomic distances between all sequenced genomes in the species complex **(** Figure 4). The ANI between the three genomes of phylotype IV strains was above 98%, with ANI between PSI07 and BDB above 99%. The ANI data between other phylotypes were also confirmed. Genome-wide ANI values above 95% are considered equivalent to the 70% DNA-DNA Hybridization (DDH) level historically used to differentiate prokaryote species [57], [58]. According to this widely-accepted standard, our results indicate that phylotype IV *R. solanacearum* strains, *R. syzygii* and BDB, which are currently described in the literature as three different species because of their phenotypic differences, belong to a single genomic species.

In the genomic era, the species definition for prokaryotes is still subject to controversy. The most accepted definition is based on the genomic similarity between organisms, and the technique of DNA-DNA hybridization, although it is not easily implemented, remains the principal tool for the determination of a bacterial species. According to the Stackebrandt committee [59], which met in 2002 to re-evaluate the bacterial species definition, “investigators are encouraged to propose new species based upon other genomic methods or techniques provided that they can demonstrate that (…) there is a sufficient degree of congruence between the technique used and DNA–DNA reassociation”. Several techniques were used to estimate the genomic similarity between bacteria in the same taxon, such as CGH microarrays [60], recombination frequency estimation [61], and MLSA [62], but genome sequencing is much more exhaustive and can greatly assist in standardizing taxonomy [57]. Using genome sequencing, Luo *et al* showed recently [63] that the omics-based methods are wholly suitable replacements for the traditional approach of defining distinctive phenotypes for new species.The Blood Disease Bacterium was initially described by Gäumann [9] as *Pseudomonas celebensis*, but this name is now invalid. Phylogenic analyses based on 16S rRNA gene sequences clustered BDB in the *R. solanacearum* species complex. Because the 16S rRNA gene sequences of *R. solanacearum* and *R. syzygii* are very similar, De Baere *et al.* proposed that *Pseudomonas syzygii* should belong to the genus *Ralstonia* [64]. In a further analysis based on DNA-DNA hybridization, Vaneechoutte *et al.* suggested that *R. solanacearum* and *P. syzygii* should be two separate species in the same genus, and officially renamed *Pseudomonas syzygii* as *Ralstonia syzygii* [65]. However, the *R. solanacearum* strain used for the DNA-DNA hybridization was the species type strain (K60, syn. LMG2299), which belongs to the phylogenetically distant phylotype IIA. We believe that the work of Vaneechoutte *et al.* would not have reached the same conclusion if a *R. solanacearum* phylotype IV strain like PSI07 had been used to represent *R. solanacearum*. The genomic and phylogenetic data on a large number of *R. solanacearum* species complex strains ([3] and this study) indicates that strains of *R. solanacearum* phylotype II, to which *R. solanacearum* strain K60 belongs, and *R. syzygii* are clearly two separate species, whereas strains of *R. solanacearum* phylotype IV and *R. syzygii* belong to the same genomic species. This conclusion is fully consistent with both overall genomic analyses and ANI comparisons.

Based on concordant results derived from several different technical approaches, the taxonomy of the *R. solanacearum* species complex should be revised, taking into account the new data from phylotype IV strains. To our knowledge, it is the first example of a species description based on genomics for any plant pathogen. According to the taxonomic rules, strains assigned to phylotype II, which includes the type strain K60^T^ ( = ATCC 11696^T^  = LMG 2299^T^) [19], should be maintained as *R. solanacearum*. Strains in phylotypes I and phylotype III form a unique genomic species, which we propose to call *Ralstonia sequeirae* sp. nov. (se.que.i'ra.e. N.L. masc. gen. n. *sequeirae*, of Sequeira, named after Luis Sequeira) with the type strain GMI1000. Strains assigned to phylotype IV also constitute a genomic species, so it is proposed to merge this group as *R. haywardii* sp. nov. (hay.war'di.i. N.L. masc. gen. n. *haywardii*, of Hayward, named after A. Christopher Hayward) with the type strain PSI07. Because *R. haywardii* sp. nov. encompasses strains with distinct phenotypes and life-styles, including *R. solanacearum*, *R. syzygii* and BDB, it is also proposed that soilborne and broad host range *R. solanacearum* strains from phylotype IV should be renamed *R. haywardii* subspecies *solanacearum* subsp. nov., proposed type strain PSI07 ( = CFBP 7288). We further propose to name the Blood Disease Bacterium that wilts banana in Indonesia as *R. haywardii* subspecies *celebensis* subsp. nov., proposed type strain R229 ( = CFBP 3568). Finally, strains of *R. syzygii* that are insect-transmitted by tube-building *Hindola spp.* cercopoids, with a host range limited to clove trees (Sumatra disease of clove) should be now called *R. haywardii* subspecies *syzygii* subsp. nov., type strain R001^T^ ( = ATCC 49543^T^ = LMG 10661^T^).

### Conclusions

The genomes of the Blood Disease Bacterium and *R. syzygii* are closely related to *R. solanacearum* strains from Indonesia, even though these highly host-specific and insect-transmitted strains are phenotypically different from broad host range *R. solanacearum* strains. Our comparative genomic analysis demonstrated that these specialized organisms, which belong to *R. solanacearum* phylotype IV, are part of the same genomic species. This reinforces our suggestion that strains in the *R. solanacearum* species complex should be divided into three genomic species [3].

The primary objective of this work was to investigate the genetic repertoire of these organisms in order to shed light upon the particular life-styles of these highly specialized organisms using comparative analyses of their genome sequence. Our data suggest that neither BDB nor *R. syzygii* has acquired substantial amounts of DNA by horizontal gene transfer from other bacteria with similar life-styles for which a genome sequence is available. The pathogenic behavior of these organisms, very unusual in the *R. solanacearum* species complex, may have resulted from ecological adaptation and genomic convergence during vertical evolution. Alternatively, the pathogenic and life-style traits may have been horizontally acquired from uncharacterized microbes. Although the genomes of BDB and *R. syzygii* are relatively large for bacterial genomes, they represent the shortest genomes within the *R. solanacearum* species complex. It is possible that this reduction in genome size may be a step in the evolution of these specialized bacteria via genome decay under selective pressures within the host. We present here phylogenic and initial comparative genomic analyses, but the critical questions about the unique biology of these two microorganisms remain to be experimentally addressed. The two genome sequences presented here will be a valuable tool for these subsequent functional studies.

## Materials and Methods

### Strains & genomic DNA extraction

Both sequenced strains were isolated in Indonesia by S. Eden-Green and maintained in the bacterial collection at Rothamstead Experimental Station (suffix R). The BDB strain R229 was isolated in 1988 as sample T389X from a wilted *Musa* hybrid banana in South Sulawesi (syn.: CFBP3568, France; RUN62, Reunion Is.; NCPPB3726, UK; UQRS465, Australia). *R. syzygii* strain R24 (syn.: CFBP6447, RUN88, UQRS466) was isolated from a diseased clove tree (*Syzygium aromaticum*) in West Java. The BDB strain R229 was assigned to phylotype IV, sequevar 10 (IV-10) together with *R. solanacearum* strain PSI07 while *R. syzygii* belongs to phylotype IV, sequevar 9 [11].

Before sequencing, virulence of these strains was confirmed as follows: *R. syzygii* strain R24 produced minute colonies after 5–7 days incubation at 29°C on modified MPW solid medium [17] containing: casein hydrolysate (7.5 g.L^−1^), sucrose (2 g.L^−1^), magnesium sulfate heptahydrate (MgSO_4_x7H_2_O; 0.25 g.L^−1^); potassium hydrogen phosphate (K_2_HPO_4_; 0.5 g.L^−1^), Agar (15 g.L^−1^), and ferric ammonium citrate (0.25 g.L^−1^). The BDB strain was cultured on Kelman's triphenyltetrazolium chloride solid medium (TZC) supplemented with 0.5 g yeast extract [66]. Bacterial suspensions in TRIS 10 mM pH 7.1 (7–9, SIGMA-ALDRICH, Saint-Louis, MO) were made from a restreaked culture of one typical colony and adjusted to 10^8^ CFU/mL as determined by measuring the optical density at 0.1 at 650 nm. Because median response time disease after transmission test with natural insect vector was 200 days [17], young clove seedlings (4–5 fully expanded leaves) were mechanically inoculated by cutting the leaves with infested scissors and banana plants were inoculated by pouring 5 ml of suspension on lateral roots that had been severed along one side using a scalpel. Negative control plants were treated with TRIS 10 mM pH 7.1 on banana plants and adjacent clove leaves. Infected plants were placed in environmental growth chambers (Rotoplan; STRADER, Pellouailles les Vignes, France). Banana pants infected with BDB developed typical wilt symptoms 10–15 days after inoculation, with reddish-brown vascular elements. Symptoms developed slowly on inoculated clove leaves (2–3 mm per 15 days), consisted of necrotic tissues with greasy water-soaked tops and yellowish margins. Control banana and clove plants did not develop any symptoms described on infected plants. Both strains were re-isolated from symptomatic plants.

Pure cultures of each strain were grown in Luria Broth liquid medium [67] at 28°C overnight. Genomic DNA was extracted using a DNeasy Blood & Tissue Kit according to the manufacturer's instructions (Qiagen, Hilden, Germany).

### Sequencing and assembly

Whole genome sequences of the BDB strain R229 and *R. syzygii* strain R24 were obtained using next-generation sequencing technologies. For BDB, we first combined 15-fold coverage 454 GSflx (Titanium version, www.roche.com) single reads with around 5-fold coverage of mate-paired 454 GSflx reads. The mate-paired library insert size was ∼3 kb. After assembly with Newbler (www.roche.com) to decrease the number of scaffolds, we added 454 GSflx (Titanium version) reads from a 9-fold coverage mate-paired library (insert size ∼8.5 kb).

The *R. syzygii* genome was sequenced directly using a mix of 454 GSflx (version Titanium) single reads and reads from a mate-paired library (insert size around 9.5 kb), which generated around 22- and 8-fold coverage, respectively. Newbler was used for the assembly of both genomes and the assembly was validated using the Consed interface [68]. The first step to organize the scaffolds and to close the replicons was to separate those believed to belong to the chromosome or to the megaplasmid molecule. So a comparison between the reference genome *Ralstonia solanacearum* GMI1000 and BDB or *R. syzygii* scaffolds more than 5 kb were done using Nucmer software [69]. After identification, the scaffolds were organized by combinatory PCRs. For this technique, oligonucleotides corresponding to scaffold extremities were selected and each primer was combined with all the others for PCR product (except those corresponding to the same scaffold). Of course, only a few results were obtained, corresponding to the correct scaffold orientation. For quality assessment, around 100-fold coverage of 36-bp Illumina reads were mapped onto the whole genome sequence, using SOAP (http://soap.genomics.org.cn), as described in [70]. Table 2 shows the main primary assembly characteristics of the two strains.

**Table 2:** Primary assembly characteristics for BDB strain R229 and *R. syzygii* strain R24.

	BDB R229	*R. syzygii* R24
Number of scaffolds	29	12
N50 scaffold size	3313414	1746931
Number of scaffolds >5 kb	5	5
Number of contigs >500	377	147
N50 contig size	25657	102346
Largest contig size	85825	364825

### Genome annotation

Automatic and expert annotation were made using the Microscope platform [71] with the same protocol as described in Remenant *et al*. [3]. Manual annotations from previously sequenced genomes of *R. solanacearum* were used to automatically annotate the BDB and *R. syzygii* strong orthologs, which were defined as protein sharing 85% identity over at least 80% of the length of the smallest protein. Any regions not automatically annotated were curated manually. Complete and assembled sequence data are publicly available via the MicroScope web interface at http://www.genoscope.cns.fr/agc/microscope/ralstoniascope. Contig sequences and annotation data have also been deposited at EMBL (http://www.ebi.ac.uk): BDB R229 [from FR854059 to FR854085] and *R. syzygii* R24 [from FR854086 to FR854092].

### Comparative genomic analyses

The Average Nucleotide Identity between genomes were calculated according Konstantinidis and Tiedje [57] and then compared with those previously published [3]. Genomic Island identification and synteny group computation were performed using the MicroScope platform [71] as described previously [3].We used the MicroScope “Gene Phyloprofile” tool to identify specific gene sets in PSI07, BDB and *R. syzygii*, with the following homology constraints: Bidirectional Best Hit, minimal alignment coverage ≥0.8, and amino-acid identity ≥30%. Graphical representation were performed using CGView software [72].

## Supporting Information

Table S1 **Overview of genomes in the** ***R. solanacearum*** **species complex.**
(XLS)

Table S2 A: percentage of CDS in synteny and average number of CDS per synteny with PSI07 as reference strain. B: PSI07 chromosome and megaplasmid alignment with the complete genome of BDB and *R. syzygii.*
(XLS)

Table S3 ***R. solanacearum*** **virulence genes (updated from Remenant** ***et al*** **** [**3**] **).**
(XLS)

Table S4 **Gene expression patterns of specific functional clusters in GMI1000 and UW551 in planta vs. in rich medium, and their presence in Phylotype IV strains PSI07, BDB, and** ***R. syzygii.***
(XLS)

Table S5 **List of adhesins and hemagluttinins genes in sequenced strains from the** ***R. solanacearum*** **species complex.**
(XLS)
